# Explicit learning based on reward prediction error facilitates agile motor adaptations

**DOI:** 10.1371/journal.pone.0295274

**Published:** 2023-12-06

**Authors:** Tjasa Kunavar, Xiaoxiao Cheng, David W. Franklin, Etienne Burdet, Jan Babič

**Affiliations:** 1 Laboratory for Neuromechanics and Biorobotics, Department of Automatics, Biocybernetics, and Robotics, Jožef Stefan Institute, Ljubljana, Slovenia; 2 Jožef Stefan International Postgraduate School, Ljubljana, Slovenia; 3 Department of Bioengineering, Imperial College of Science, Technology and Medicine, London, United Kingdom; 4 Neuromuscular Diagnostics, Department Health and Sport Sciences, TUM School of Medicine and Health, Technical University of Munich, Munich, Germany; 5 Munich Institute of Robotics and Machine Intelligence (MIRMI), Technical University of Munich, Munich, Germany; 6 Munich Data Science Institute (MDSI), Technical University of Munich, Munich, Germany; Kennedy Krieger Institute/Johns Hopkins University School of Medicine, UNITED STATES

## Abstract

Error based motor learning can be driven by both sensory prediction error and reward prediction error. Learning based on sensory prediction error is termed sensorimotor adaptation, while learning based on reward prediction error is termed reward learning. To investigate the characteristics and differences between sensorimotor adaptation and reward learning, we adapted a visuomotor paradigm where subjects performed arm movements while presented with either the sensory prediction error, signed end-point error, or binary reward. Before each trial, perturbation indicators in the form of visual cues were presented to inform the subjects of the presence and direction of the perturbation. To analyse the interconnection between sensorimotor adaptation and reward learning, we designed a computational model that distinguishes between the two prediction errors. Our results indicate that subjects adapted to novel perturbations irrespective of the type of prediction error they received during learning, and they converged towards the same movement patterns. Sensorimotor adaptations led to a pronounced aftereffect, while adaptation based on reward consequences produced smaller aftereffects suggesting that reward learning does not alter the internal model to the same degree as sensorimotor adaptation. Even though all subjects had learned to counteract two different perturbations separately, only those who relied on explicit learning using reward prediction error could timely adapt to the randomly changing perturbation. The results from the computational model suggest that sensorimotor and reward learning operate through distinct adaptation processes and that only sensorimotor adaptation changes the internal model, whereas reward learning employs explicit strategies that do not result in aftereffects. Additionally, we demonstrate that when humans learn motor tasks, they utilize both learning processes to successfully adapt to the new environments.

## I. Introduction

To adapt our motions to changes in the environment our central nervous system mostly utilizes sensory prediction error and reward prediction error [1–4]. The process of learning through sensory prediction error is known as sensorimotor adaptation, whereas learning that leverages reward prediction error is referred to as reward learning. The basis for the sensory prediction error is the sensory consequence of motion, observed by our sensory organs such as vision and proprioception. Sensorimotor adaptation is a process that allows alterations to the internal model either through a feedforward model, which maps motor commands to corresponding sensory consequences [5, 6], or through policy learning [7]. These alterations to the internal model occur without conscious awareness and can result in large and prolonged aftereffects, reflecting changes made to the internal model during prior adaptation [5, 8]. Reward prediction error is the difference between internal reward prediction and the actual observed reward [2, 9]. The basis for the reward prediction error is the reward consequence, a subjective measure of usefulness, modulated by dopamine through reinforcement learning [1, 10].

Motor learning can rely either on sensory prediction error, reward prediction error, or both. When sensory feedback is available, adaptation is predominantly driven by sensory prediction error [1], while in the case of limited sensory information, motor learning is driven by reward prediction error [4, 11]. Studies have demonstrated that relying on either type of prediction error can yield similar levels of motor adaptation [1, 12].

For example, when hitting the ball in tennis, you can observe the sensory consequences of your swing, like the position of your arm, through vision or proprioception during the swing. The sensory prediction error arises when the sensory information does not align with your expected motion. This error results from the neural noise or external factors like a changed racket weight. Sensorimotor adaptation accounts for this error by making alterations to the internal model without our conscious awareness. Furthermore, success or failure in hitting the ball translates to a reward consequence, expressed as a reward prediction error. A successful hit means you will likely repeat the same motion, however, a miss might lead you to consciously adjust your swing. Reward prediction error proves particularly useful when dealing with a changing environment, such as strong wind during a tennis match. If there is a wind, you might adjust your swing towards the opposite direction to compensate for the wind resistance. In the real world, there are many such parameters that attribute to the changing environment and the human motor control system is especially good in dealing with such randomly changing perturbations, which affect our movements.

Based on previous studies showing that sensorimotor learning can result in large and prolonged aftereffects reflecting the changes to the internal model during previous adaptation, we hypothesize that learning based on sensory prediction error might limit the ability of subjects to deal with randomly changing perturbations. Specifically, sensorimotor adaptation to a particular perturbation during an earlier task will not help subjects in the task where perturbations vary randomly. On the other hand, subjects learning from reward consequences would use explicit learning strategies that would not produce aftereffects. We hypothesize that learning based on reward prediction error would allow subjects to adapt quicker to randomly changing perturbations. To test these hypotheses, we used a visuomotor paradigm [13–15] where the goal of the motor task is to hit a designated target region with a visual cursor controlled by the motion of a hand. During the learning phase, an angular rotation is imposed on the cursor and subjects have to learn to counter the perturbation by moving the hand in the opposite direction [16]. Moreover, to differentiate between reward and sensory prediction errors, we modified the task as in [1, 12] by presenting subjects either the information on sensory consequences of their movement, signed end-point error or just binary reward.

To further explain how sensorimotor adaptation and explicit reward learning are combined to drive motor adaptations we designed a computational model that combines both motor learning processes. Even though we can separate the sensorimotor adaptation and explicit reward learning processes in human motor experiments fairly well, we cannot guarantee a perfect separation due to the inability to fully isolate the two processes or completely inhibit one of them. For this reason, we investigated the individual contributions of either sensorimotor adaptation or explicit reward learning on its own by simulating them separately with a computational model. We built an iterative learning control model with modules that differentiate between sensory and reward prediction errors. This allowed us to get an insight into how these two learning processes are integrated into motor control. The model successfully replicates the experimental results, validating that it effectively captures these learning processes. Moreover, the sensorimotor adaptation and explicit reward learning processes explain the differences in aftereffects and the ability to switch between perturbations.

## II. Results

**Fig 1:**
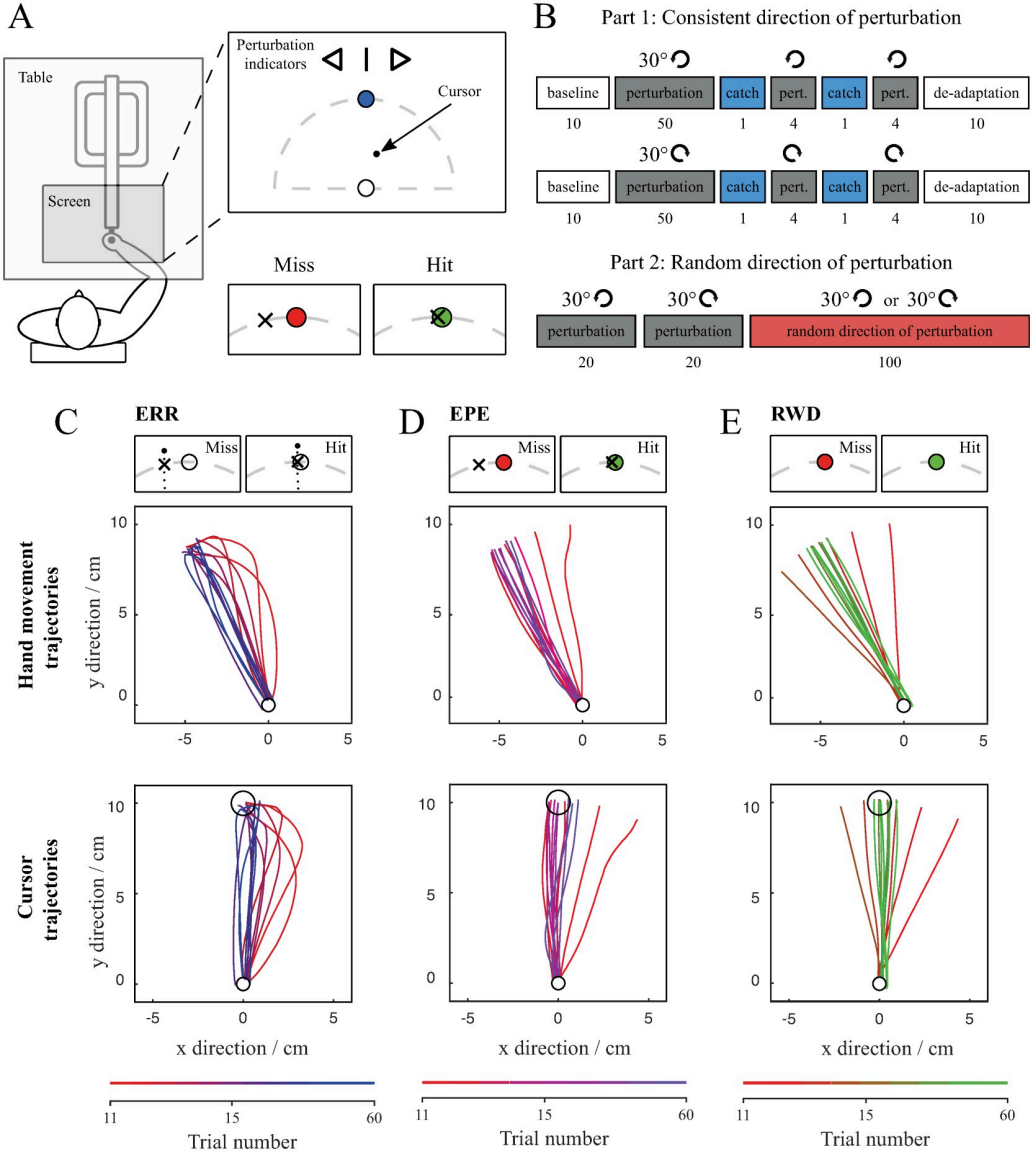
Experimental setup (A). Subject is holding a handle of the Haptic Master covered with a screen. Top right figure shows a screen with a start position, target, cursor, and perturbation indicators. Bottom right figure shows indicators of a missed or hit trial. Experimental protocol (B). The experiment is divided into two parts: in the first part the direction of perturbation is consistent whereas in the second part the direction of perturbation is random. White blocks represent trials without any perturbation. Grey blocks represent trials with a consistent perturbation in one direction, while the red block represents trials where the direction of perturbation is random. Blue blocks represent trials when the perturbation was omitted (catch trials). Arrows show the direction of perturbation and numbers below the blocks denote the number of trials per specific block. The order of the perturbation direction (clockwise or counter clockwise) was counterbalanced across subjects. Example trajectories for the ERR (C), EPE (D) and RWD (E) groups. Figure shows cursor and hand movement trajectories of three representative subjects for the initial five perturbed trials followed by every sixth trial. The progression of the trials is represented with the transition of the red into blue colour for the ERR group, red into violet colour for the EPE group and red into green colour for the RWD group.

Subjects were asked to adapt to visuomotor rotation of 30° in the clockwise or counter-clockwise direction while reaching towards a target with their right hand (Fig 1A). Before each trial, perturbation indicators in the form of visual cues were presented to inform the subjects of the presence and direction of the perturbation. Their task was to hit the target when it appeared on the screen in front of them. After each motion, a cross mark showed the location where the hand passed the distance of the target. Additionally, the target changed its colour to green if it was hit or to red if it was missed. There are two types of motor errors present during this kind of motion: sensory prediction error which is defined as the difference between the expected and the observed position of the hand during the motion (represented as the location of the cursor), and reward prediction error which is defined as the difference between the expected and the observed success of the motion. To dissociate these two types of errors, we assigned subjects into three groups based on the error information that was provided to them. First group received full visual feedback to study the influence of the sensory prediction errors throughout the reaching movement (ERR group). The second group received end-point error information at the end of the movement (EPE group). Compared to the binary reward, the end-point error in the EPE group was meant to emulate a more realistic situation in our daily lives, where we usually know the size and direction of our mistakes. It also avoided the very extensive exploration needed in the case of a binary reward. The third group received only binary reward feedback at the end of the movement (RWD group) as the reward prediction error. The reward feedback was given immediately after a trial was completed. Subjects first learned to adapt to clockwise and counter clockwise perturbations separately and then proceeded with the randomly changing direction of the perturbation (Fig 1B).

During the initial baseline movements, when there was no perturbation, all the ERR, EPE and RWD groups exhibited comparable movements with nearly straight-line hand and cursor trajectories and the Cursor path area values close to zero. The average Cursor path area of baseline trials was -0.22 ± 3.65 cm^2^ for the ERR group, 1.18 ± 4.56 cm^2^ for the EPE group and 0.80 ± 5.28 cm^2^ for the RWD group. Statistical analysis showed no significant difference between the groups (F_23_ = 0.20, p = .817). Average Cursor angle of baseline trials was -1.45 ± 7.28° for the ERR group, 2.05 ± 6.26° for the EPE group and 0.80 ± 5.28° for the RWD group, with no significant difference between the groups (F_23_ = 0.63, p = .542).

### A. Part 1: Consistent direction of perturbation

The aim of the first part of the experiment was to investigate the adaptation to visuomotor perturbation and to explore the possible differences in motor learning between the ERR, EPE and the RWD groups. All subjects received the information about the direction of the perturbation in the form of visual cues before the start of each trial. ANOVA analysis showed groups differed in the time needed to perform the movement (F_23_ = 9.28, p = .001). Post-hoc t-tests further showed that on average, the ERR group needed more time to perform the movements (1.09 ± 0.31 s) compared to the EPE group (0.63 ± 0.16 s; t_14_ = 3.70, p = .002) and RWD group (0.70 ± 0.19 s; t_14_ = 3.05, p = .007).

When the perturbation was first introduced, the hand movement trajectories remained straight while the cursor trajectories shifted in the same direction as the perturbation. Fig 1C–1E shows the hand movement trajectories and cursor trajectories for the ERR, EPE and RWD groups. It should be noted that the EPE and RWD groups did not see the actual cursor during the experiment. In the ERR group, initial cursor trajectories were curved and subjects still managed to hit the target (Fig 1C), presumably by using visual feedback during the movement. After a few trials, cursor trajectories became notably less curved as subjects proceeded to learn. In contrast to the ERR group, the initial cursor trajectories of the EPE and RWD groups were almost straight but rotated and with a larger end-point error (Fig 1D and 1E). During the adaptation, subjects kept the cursor trajectories straight, but changed the direction of movement which decreased the end-point error. While subjects in the EPE group saw the end-point error and could change the direction of the movement accordingly, the subjects in the RWD group had to explore the movement space to find a movement where they hit the target.

**Fig 2:**
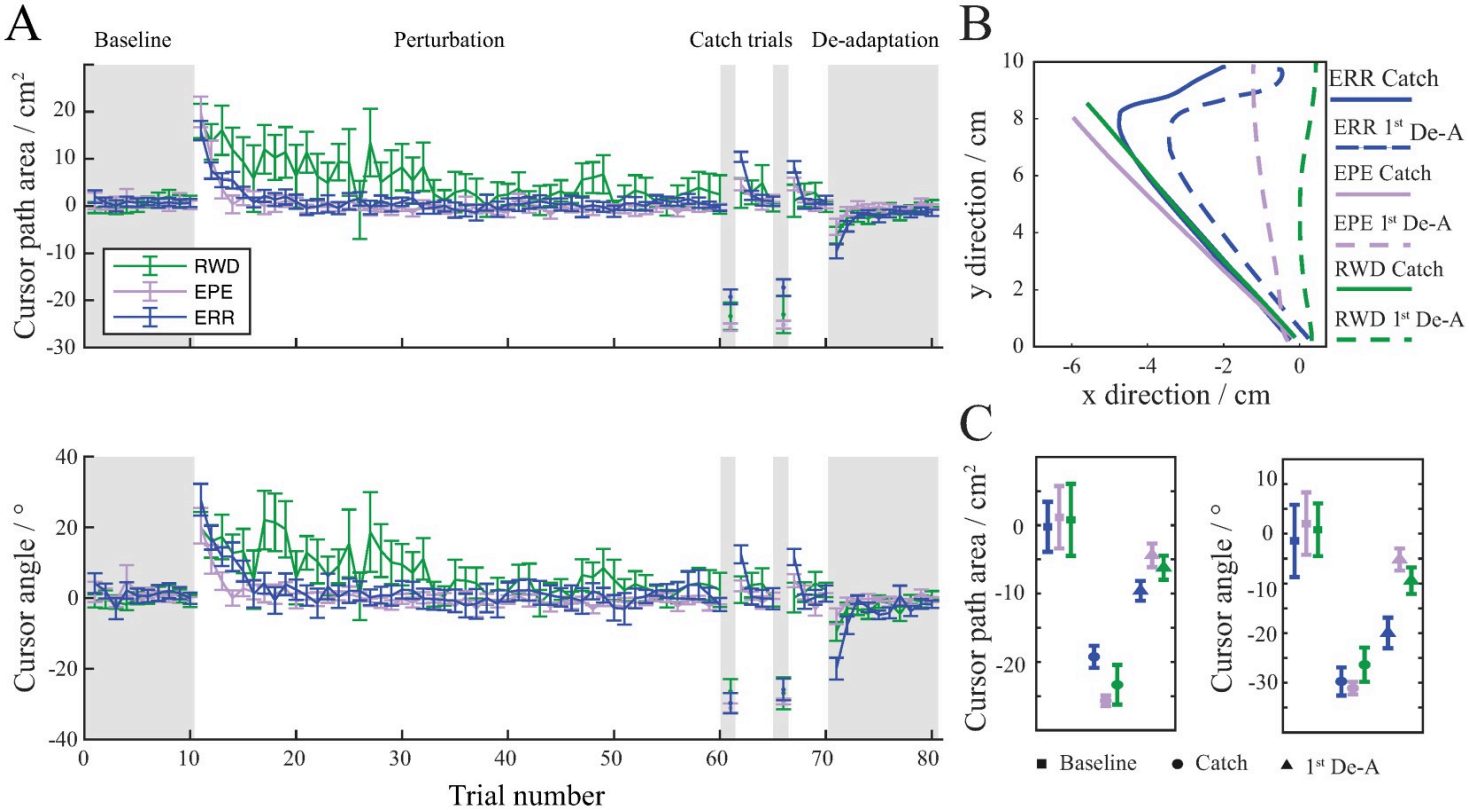
Movements during consistent direction of perturbation. (A) Mean values of Cursor path area and Cursor angle combined for both directions of perturbation showing the progression of learning. Cursor path area and Cursor angle for the ERR group are shown in blue, for the EPE group in violet and for the RWD group in green. Error bars show standard error of the mean. Shaded areas represent trials without perturbation and non-shaded areas represent trials with perturbation. (B) Example trajectories of catch and de-adaptation trials. Because there is no perturbation for these trials, cursor trajectories correspond to the hand movement trajectories. Trajectories from the ERR group are shown in blue, for the EPE group in violet and for the RWD group in green. (C) Comparison of aftereffects between the ERR, EPE and RWD groups. Average Cursor path area and Cursor angle for baseline, catch and de-adaptation trials. Error bars show standard deviation.

Subjects in all groups successfully adapted to visuomotor rotation, which can be seen as a decrease in Cursor path area during perturbation trials (Fig 2A). In effect, cursor trajectories became similar to the baseline cursor trajectories with Cursor path area and Cursor angle coming close to zero for all groups.

To assess learning, we looked at the Cursor path area of catch trials when the perturbation was absent. Absolute Cursor path area values of catch trials were comparable between all groups. There was no significant difference between groups for the first (F_47_ = 2.73, p = .076) as well as the second catch trial (F_47_ = 2.58, p = .087). Since the ERR group was provided with sensory information and was able to correct their movements during a single movement, we additionally looked at the initial part of the movement (Cursor angle), before the contribution of the feedback. During the catch trials, the initial part of the arm reaching movements was similar for subjects in all groups and there was no difference between the corresponding Cursor angle for neither the first catch trial (F_47_ = 0.82, p = .448) nor for the second catch trial (F_47_ = 0.29, p = .753).

To assess the after-effects of learning, we looked at the 1^st^ de-adaptation trial. The perturbation was removed for de-adaptation trials. Contrary to the catch trials, subjects were informed of the lack of perturbation during de-adaptation trials. However, visual information during the trial was the same as in previous trials and there was no difference between catch trials and de-adaptation trials, except for the difference in visual cues. There was an apparent difference in Cursor angle between the 1^st^ de-adaptation trials of all groups (F_46_ = 7.99, p = .001). The average value of Cursor angle for the 1^st^ de-adaptation trial of group ERR was significantly larger (in absolute terms) than the Cursor angle for the 1^st^ de-adaptation trial of the group EPE (t_30_ = 3.89, p = .001) and RWD (t_29_ = 2.52, p = .028). Subjects in the ERR group had a large aftereffect with curved trajectories similar to those during the catch trials (t_15_ = 1.39, p = .188) (Fig 2B and 2C). Since there was no perturbation present during the 1^st^ de-adaptation trials and catch trials, cursor trajectories correspond to hand movement trajectories. The average value of Cursor angle for the 1^st^ de-adaptation trial was significantly larger (in absolute terms) than the average value of Cursor angle for the baseline trials (t_15_ = 6.96, p < .001). On the other hand, subjects in the groups EPE and RWD exhibited smaller aftereffect during the 1^st^ de-adaptation trial (t_15_ = 10.53, p < .001 for the EPE group and t_14_ = 3.05, p = .008 for the RWD group). Their trajectories were closer to those during the baseline trials (Fig 2B and 2C), however, the Cursor angles of 1^st^ de-adaptation trials were still significantly different from Cursor angles of baseline trials (t_15_ = 3.07, p = .008 for the EPE group and t_13_ = 3.10, p = .008 for the RWD group).

### B. Part 2: Random direction of perturbation

**Fig 3:**
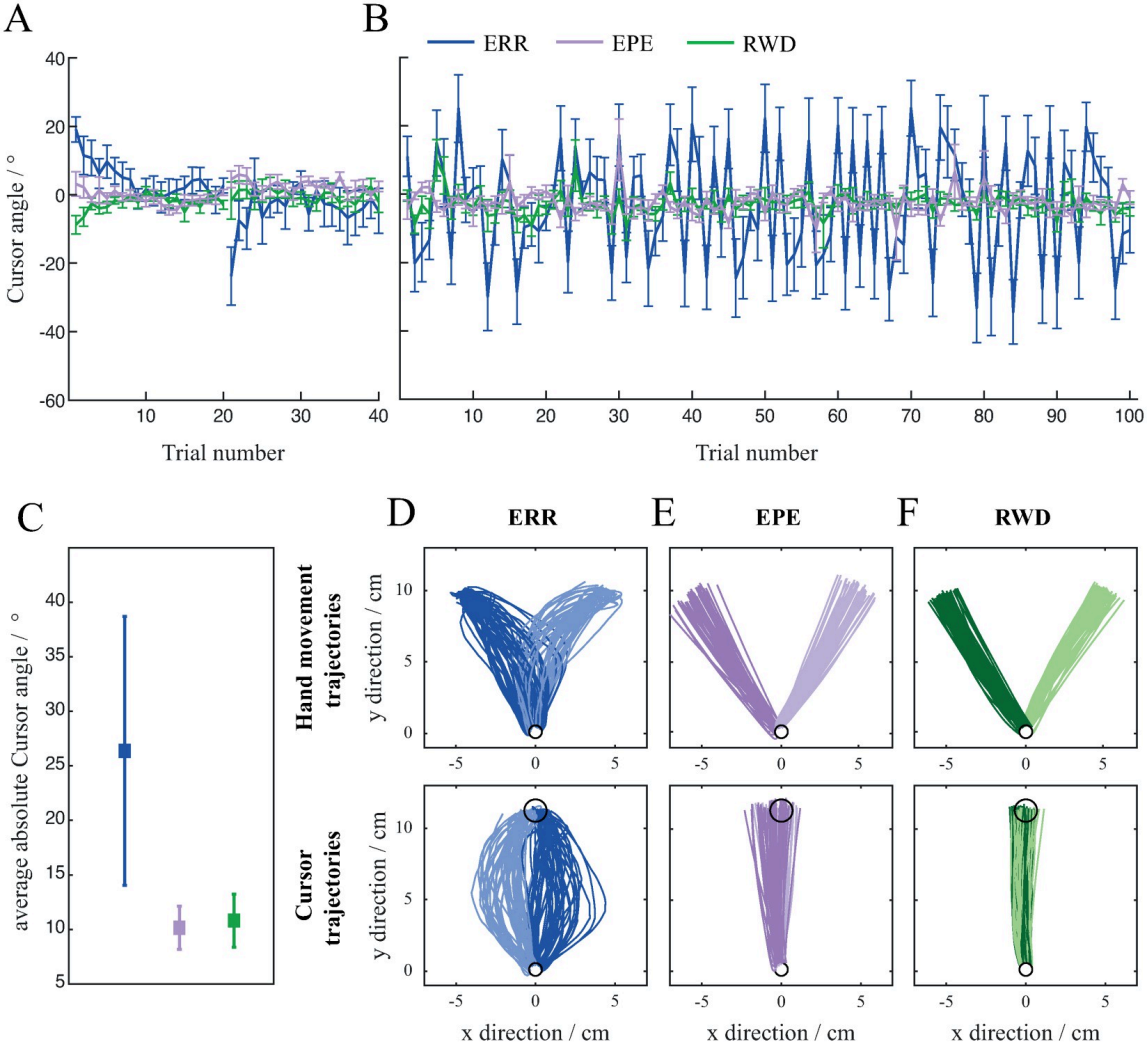
Movements during random direction of perturbation. Mean Cursor angle during short block of consistent direction of perturbation (A) and random direction of perturbation (B). Mean Cursor angle is presented as a function of trials. Cursor angle for the ERR group is shown in blue, for the EPE group in violet and for the RWD group in green. Error bars show standard error of mean. (C) Cursor trajectories of a subject from the ERR group. Light blue denotes trajectories with perturbation in the left direction and dark blue denotes trajectories with perturbation in the right direction. (D) Cursor trajectories of a subject from the EPE group. Light violet denotes trajectories with perturbation in the left direction and dark violet denotes trajectories with perturbation in the right direction. (E) Cursor trajectories of a subject from RWD group. Light green denotes trajectories with perturbation in the left direction and dark green denotes trajectories with perturbation in the right direction.

In the second part of the experiment, subjects were presented with a randomly changing direction of the perturbation to investigate how the two different prediction errors affect the ability to successfully perform the motion when the direction of perturbation switches from trial to trial. Subjects first performed 20 trials with clockwise perturbation and 20 trials with counter clockwise perturbation (Fig 3A) and then proceeded to the random direction of perturbation (Fig 3B). The sequence of the trials was random; however, all subjects received the information about the direction of the perturbation in the form of visual cues before the start of each trial. We wanted to see if subjects can successfully adapt their movements to two opposing perturbations based on the advance information about perturbation. The cursor trajectories of subjects in the ERR group were similar to the initial cursor trajectories from the first part of the experiment when subjects first experienced the perturbation and had not yet adapted their motor behaviour. Their cursor trajectories were curved to the left when there was a counter clockwise rotation (light blue on Fig 3D) and to the right when there was a clockwise rotation (dark blue on Fig 3D). In stark contrast, subjects in the EPE and RWD groups could easily adapt to the perturbation that was randomly changing in direction. Their cursor trajectories were almost straight (light and dark violet lines for the EPE group and light and dark green lines for the RWD group on Fig 3E and 3F) with no apparent difference between the two directions of perturbation.

Even though subjects in all groups had previously learned to counteract the perturbation in opposite directions, only the EPE and RWD groups were able to deal with the perturbation in random direction (Fig 3). Subjects in the ERR group made large errors and were not able to adapt to changing direction of perturbation, which is reflected in higher Cursor angle values. On the other hand, subjects in the EPE and RWD groups made smaller errors and were able to adapt to the changing direction of perturbation, which is reflected in smaller Cursor angle values that are closer to zero. There was a significant difference in mean Cursor angle between the groups (F_23_ = 12.49, p < .001). Mean Cursor angle of the ERR group was significantly larger than the mean Cursor angle of the EPE group (t_14_ = 3.67, p < .001) as well as the RWD group (t_14_ = 3.50, p = .001). There was also a difference in inter subject variance between groups (K^2^(2) = 25.04, p < .001). Variance in ERR group was higher compared to the variance in the EPE (F_1_ = 25.83, p < .001) and the RWD groups (F_1_ = 38.93, p < .001).

During the 100 trials with random direction of perturbation, there was no additional adaptation to the alternating perturbations. In both groups, Cursor angle of the first 10 trials was not significantly different as Cursor angle of the last 10 trials (t_14_ = 0.08, p = .936, t_14_ = 0.50, p = .626, t_14_ = 1.09, p = .293 for the ERR, EPE and RWD group respectively).

### C. Simulation results

**Fig 4:**
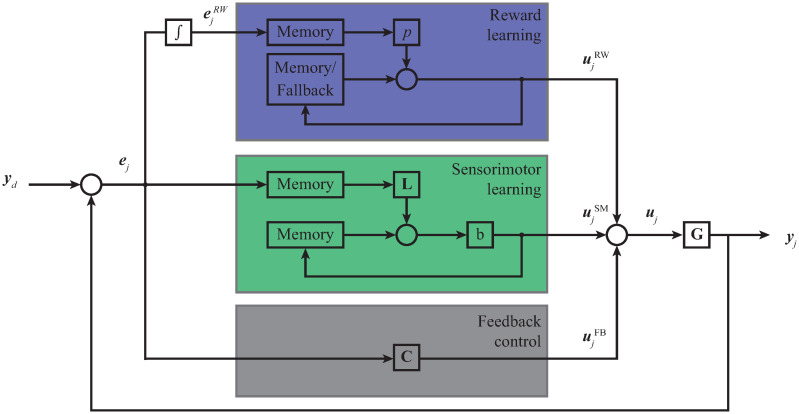
Architecture of the computational model. The parallel architecture computes the control signal ***u***_*j*_ as a sum of control signals that are based on explicit reward learning $u_{j}^{RW}$, sensorimotor adaptation $u_{j}^{SM}$ and feedback controller $u_{j}^{FB}$. It directly generates the motor commands to the plant G. The integrator block ∫ represents the calculation of the reward error $e_{j}^{RW}$ which can be end-point error or binary information. Fallback module represents the initial conditions of motor commands for the first simulation trial and memory module represents motor commands from the previous simulation trial.

To investigate the individual characteristics and contributions of either sensorimotor adaptation or explicit reward learning to the overall motor learning, we designed a computational model which distinguishes between the two learning processes. By using the simulation, we were able to analyse sensorimotor adaptation and explicit reward learning processes separately and make comparisons between their outcomes. We modelled the experimental system of a human arm reaching towards a target where the arm was represented as a point mass. The system state represented position and velocity in a polar coordinate system. Performance of the system was determined by the difference between the desired trajectory and actual trajectory and included errors in both position and velocity. To model the human learning process, we used a non-causal iterative learning control (ILC) system, where a current-iteration feedback controller is incorporated with ILC in the parallel architecture and combined with learning based on reward prediction error as seen on Fig 4.

Sensorimotor adaptation was based on sensory prediction error throughout the movement and explicit reward learning was based on reward prediction error at the end of the movement. Here we distinguished between the reward in the form of end-point error (as in the group EPE) and a binary reward (as in the group RWD). The previously mentioned learning processes were first simulated separately; sensorimotor adaptation regarded all the sensory information during the trial and included feedback control, while explicit reward learning disregarded sensory information during the trial and did not include feedback control. Furthermore, the sensorimotor adaptation and explicit reward learning processes were then combined to simulate the learning observed in experimental study. Sensorimotor adaptation was combined with different rates of explicit reward learning where end-point error was taken as a reward prediction error. Following, the end-point error learning without feedback control was combined with different rates of sensorimotor adaptation. Simulation mimicked the experimental design and included 80 trials comprising of baseline, perturbation, catch and de-adaptation trials with 40 time steps of 0.02s each. The system had a visuomotor rotation feature, where the angle of rotation was determined by the angle *β*. During the simulation, initial trials had *β* set to 0. For the following trials, the angle *β* was set to 30°, except for the two catch trials and de-adaptation trials, where *β* was again set to 0.

**Fig 5:**
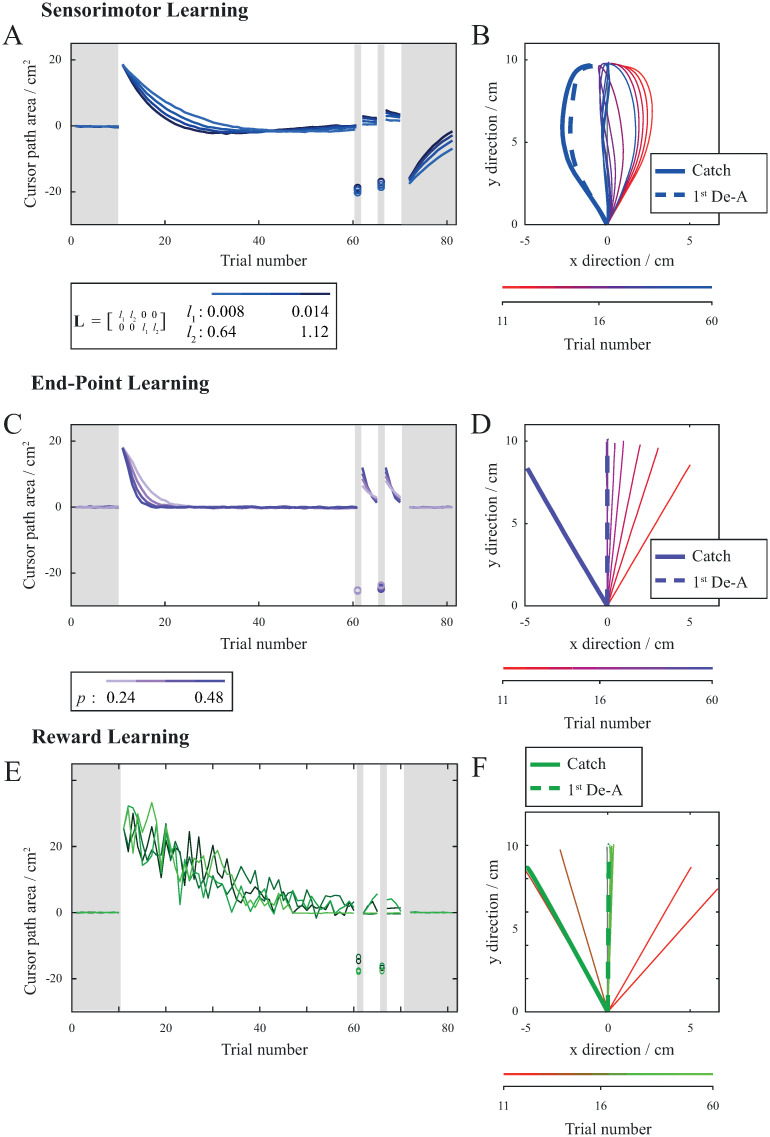
Simulation of sensorimotor adaptation (A-B) end-point error learning (C-D) and binary reward learning (E-F). (A) Mean values of Cursor path area showing the progression of sensorimotor adaptation. Different shades of blue represent different sensorimotor adaptation parameter values in matrix **L**. Shaded areas represent trials without perturbation and non-shaded areas represent trials with perturbation. (B) Example of simulated cursor trajectories for sensorimotor adaptation showing the initial four perturbed trials followed by every sixth trial, catch trial and 1^st^ de-adaptation trial. The progression of the trials is represented with the transition of the red into blue colour. Bold blue line depicts catch trial and bold dashed blue line depicts the 1^st^ de-adaptation trial. (C) Mean values of Cursor path area showing the progression of end-point error learning. Different shades of violet represent different values of *p* for explicit reward learning. Shaded areas represent trials without perturbation and non-shaded areas represent trials with perturbation. (D) Example of simulated cursor trajectories for end-point error learning showing initial four perturbed trials followed by every sixth trial, catch trial and 1^st^ de-adaptation trial. The progression of the trials is represented with the transition of the red into violet colour. Bold violet line depicts the catch trial and bold dashed violet line depicts the 1^st^ de-adaptation trial. (E) Mean values of Cursor path area showing the progression of binary reward learning. Different shades of green represent different exploration runs. Shaded areas represent trials without perturbation and non-shaded areas represent trials with perturbation. (F) Example of simulated cursor trajectories for binary reward learning showing initial four perturbed trials followed by every sixth trial, catch trial and 1^st^ de-adaptation trial. The progression of the trials is represented with the transition of the red into green colour. Bold green line depicts the catch trial and bold dotted green line depicts the 1^st^ de-adaptation trial.

We first simulated sensorimotor adaptation based on sensory prediction error (Fig 5A and 5B), without taking into account the reward prediction error. This was done by setting the parameter *p*, which determines explicit reward learning, to zero and modulating parameters in the matrix **L**, which determines sensorimotor adaptation. Trajectories were updated from one simulation trial to the next based on the errors in position and velocity at every time-step. We also included input from feedback control which was updated from one time-step to the next during a single simulation trial. Initial trials without the perturbation resulted in straight cursor trajectories, since the model has previous motor knowledge for an unperturbed movement stored in the memory / fallback module. When *β* was set to 30° the cursor trajectory shifted in the right direction at the beginning of the trial and then moving closer to the middle as a result of the feedback controller. After a few trials, the model learns to adapt to the perturbation and Cursor path area decreases over trials. The slope of the decline depends on the different values of the parameters in **L** that affect the learning rate. During the catch trials, where *β* was set to 0, the same motor commands were used as in the previous trials with perturbation. This resulted in large aftereffect (bold blue trajectory on Fig 5B). During the de-adaptation trials (dotted blue line on Fig 5B), *β* was again set to 0 which resulted in similar aftereffect as observed during catch trials.

We then modelled explicit reward learning based on end-point error (Fig 5C and 5D) without sensorimotor adaptation and feedback control. Learning based on reward prediction error was used to plan the direction of movement. The sensory information during the trial was disregarded and only the information on the error at the final time step of the previous simulation trial was considered. This was done by setting all the parameters in matrix **L**, which determines sensorimotor adaptation, to zero and modulating parameter *p*, which determines explicit reward learning. As in the case of sensorimotor adaptation, initial trials without the perturbation resulted in straight cursor trajectories. When *β* was set to 30°, the cursor trajectory shifted in the right direction without any corrections during the trial, resulting in a straight but angled cursor trajectory. After a few trials, the model learned to adapt to the perturbation and Cursor path area decreases over trials. The slope of the decline depends on the different values for parameter *p* that affect the learning rate. Based on the end-point error, the model adjusted the direction of movement and planned the trajectory accordingly. During catch trials, where *β* was set to 0, the same motor commands were used as in the previous trials with perturbation. During catch trials, the planned trajectory was based on the previously learned perturbation and therefore resulted in significant aftereffects (bold violet trajectory on Fig 5D). On the other hand, in the de-adaptation trials, the new motion was based on the motor commands for the straight movement that were stored in the fallback module. Since there were no changes due to the sensorimotor adaptation and the changes resulting from explicit reward learning were disregarded due to the new information on the perturbation, the direction of movement was adjusted accordingly. During the de-adaptation trials (dashed violet line on Fig 5D), *β* was again set to 0 which resulted in a straight trajectory similar to the initial trials without perturbation.

Following, we modelled explicit reward learning based on binary error (Fig 5E and 5F) without sensorimotor adaptation and feedback control. Similarly as in end-point learning, there was no sensory information provided during the simulated trials resulting in no contribution from the feedback controller. Additionally, there was no information about the end-point error. The only information provided was, if the trial was a success or a failure. If the trial was a success, motor commands were stored and used in the next trial. If the trial was a failure, we used exploration to determine the movement in the next simulation trial. In this case, parameter *p* was randomly determined from a uniform distribution to allow for exploration. Initial trials without the perturbation resulted in straight cursor trajectories. When *β* was set to 30° the cursor trajectory shifted in the right direction without any corrections during the trial, resulting in a straight but angled cursor trajectory, similar as in end-point learning. This was followed by multiple exploration trials before model found a solution. After initial period of exploration, a successful solution is found and Cursor path area decreases over trials. Similar as in end-point error learning, the planned trajectory during catch trials was based on the previously learned perturbation and therefore resulted in significant aftereffects (bold green trajectory on Fig 5F). De-adaptation trials (dotted green line on Fig 5F) resulted in a straight trajectory similar to the initial trials without perturbation.

**Fig 6:**
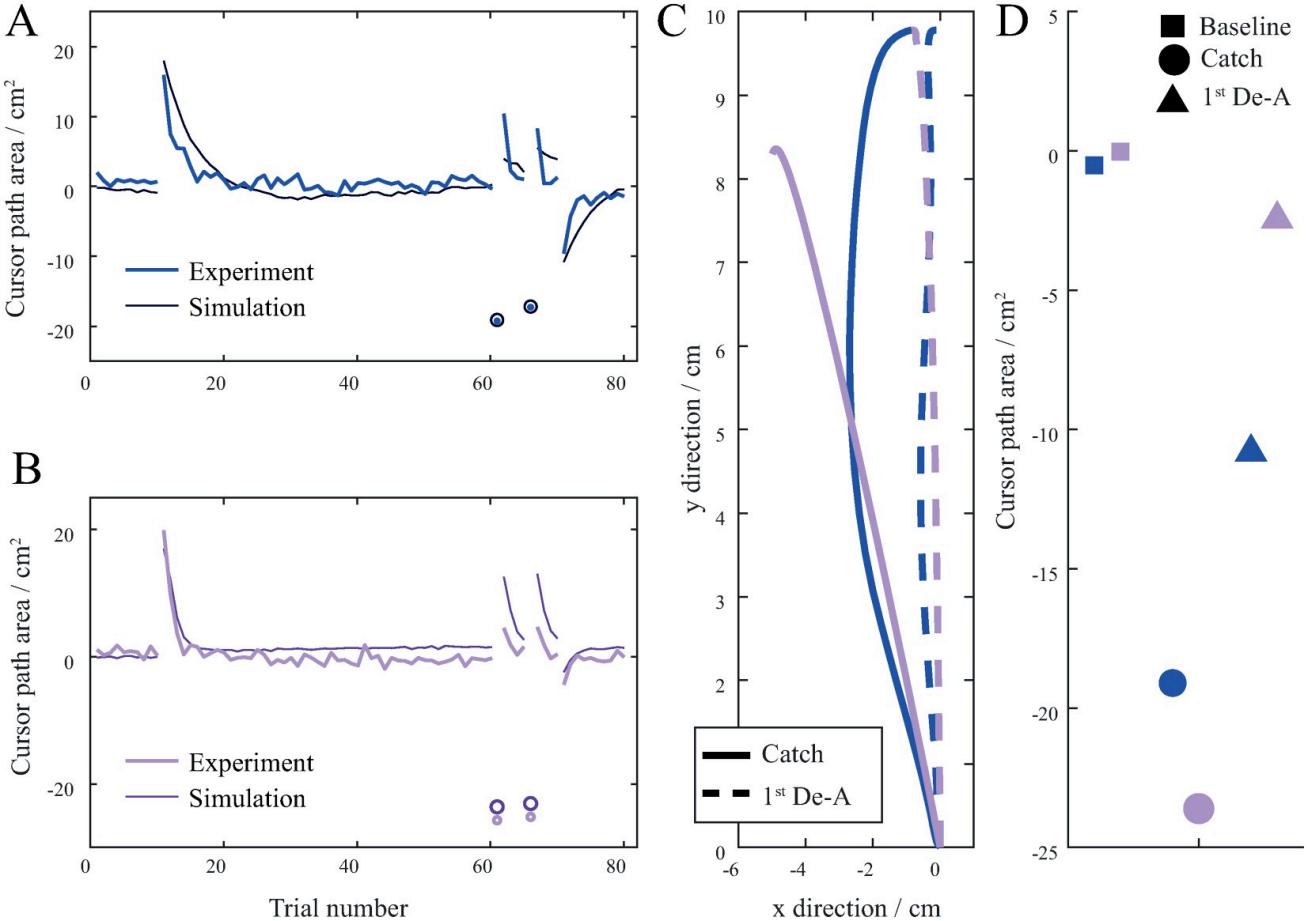
Simulation of a combination of sensorimotor and explicit reward learning. (A) Mean values of Cursor path area showing the progression of learning simulating results from the ERR group. Black represents simulation results and blue represents experimental results. (B) Mean values of Cursor path area showing the progression of learning simulating results from the EPE group. Dark violet represents simulation results and light violet represents experimental results. (C) Example of simulated trajectories showing the catch trials and 1^st^ de-adaptation trials. Because there is no perturbation for these trials, cursor trajectories correspond to hand movement trajectories. Bold blue line depicts catch trial and bold dashed blue line depicts 1^st^ de-adaptation trial for ERR group. Bold violet line depicts catch trial and bold dashed violet line depicts 1^st^ de-adaptation trial for RWD group. (D) Average Cursor path area for baseline, catch and de-adaptation trials.

We then proceeded to simulate the results obtained in the experimental study, by combining the explicit reward learning and sensorimotor adaptation. Two different simulations were done, one with the sensory information provided throughout the trials as in the ERR group and the other without the sensory information and only with the end-point error information provided at the end of the trials as in the EPE group. Since the goal here was to simulate the experimental results, the parameter values used for both sensorimotor adaptation and explicit reward learning were chosen so that the simulation results best match the result from the experiment. We first modelled learning with sensory information provided (Fig 6A). Parameters in matrix **L**, which determines sensorimotor adaptation were set to $\left[\begin{array}{cccc} 0.02 & 0.9 & 0 & 0\\ 0 & 0 & 0.02 & 0.9 \end{array}\right]$ and parameter *p*, which determines explicit reward learning, was set to 0.36. Trials during perturbation and catch trials were similar as in previous simulation. However, Cursor path area values for de-adaptation trials were smaller as in previous simulation, indicating smaller aftereffects when the absence of perturbation was known. The aftereffects were influenced by the amount of explicit reward learning present. Without explicit reward learning the aftereffects were similar to the ones observed during the catch trials, while with higher learning rates of explicit reward learning the aftereffects decreased.

We then modelled learning without sensory information provided (Fig 6B). Parameter *p*, which determines explicit reward learning, was set to 0.5 and parameters in matrix **L**, which determines sensorimotor adaptation were set to $\left[\begin{array}{cccc} 0.0012 & 0.96 & 0 & 0\\ 0 & 0 & 0.0012 & 0.096 \end{array}\right]$. Trials during perturbation and catch trials were similar as in previous simulation. However, Cursor path area values for the de-adaptation trials were higher as in the previous simulation, indicating higher aftereffects when the absence of perturbation was known. The aftereffects were influenced by the amount of sensorimotor adaptation present. Without the sensorimotor adaptation the aftereffects were not present, while with the higher learning rates of sensorimotor adaptation the aftereffects increased. Fig 6C and 6D shows example trajectories of catch and de-adaptation trials and average Cursor path area for baseline, catch and de-adaptation trials.

## III. Discussion

To compare and analyse learning based on sensory prediction error versus learning based on reward prediction error, we used a visuomotor paradigm where subjects performed arm reaching movements and were presented either with the information on sensory consequences of their movement or reward consequence of their movement. To further understand the contributions of sensorimotor adaptation and explicit reward learning to the overall adaptation to visuomotor rotation, we designed a computational model which distinguishes between learning based on sensory prediction error and reward prediction error.

The aim of the first part of the experiment was to investigate the adaptation to visuomotor perturbations and to explore the possible differences between the sensorimotor adaptation, end-point error learning and binary reward learning. Subjects could successfully adapt to visuomotor rotation in all cases, i.e., relying either on sensory or on reward prediction error. There were similar levels of motor adaptation, observed through comparable Cursor path area and Cursor angle during adaptation, and similar catch-trial aftereffects. This is consistent with the findings of previous studies [1, 12] showing that motor learning can rely on any form of prediction error and still lead to similar levels of motor adaptation.

De-adaptation trials revealed differences in the aftereffects. As expected from previous works [8, 17], learning based on sensory prediction error results in large and prolonged aftereffects. This was observed in the process of de-adaptation where there was an apparent difference between the base trials and the 1^st^ de-adaptation trials. These apparent aftereffects suggest that subjects in the ERR group used sensorimotor adaptation to alter their internal model during the adaptation. On the other hand, explicit learning based on reward prediction error did not produce aftereffects as with sensorimotor adaptation. Subjects did not exhibit any aftereffect during the 1^st^ de-adaptation trial and their trajectories resembled baseline trials. This coincides with previous work, where they showed that reward information alone failed to induce sensorimotor recalibration [3, 4]. However, this might only apply to instances where only one target is used in the experimental setup, since it was shown that learning based on end-point error produces higher aftereffects than observed in our study in cases where multiple targets are presented [17]. This might be because explicit strategies are harder to implement in cases of multiple targets.

To investigate how the two different prediction errors affect the ability to successfully perform the motion when the direction of perturbation switches from trial to trial, subjects were presented with a randomly changing direction of perturbation. Even though subjects relying on sensory prediction error had previously learned to counteract the perturbation in both directions, they could not deal with the perturbation in random direction. This suggests that they could not make timely changes to the internal model and distinguish between motor commands obtained for a specific perturbation. This is in line with previous research, which showed that sensorimotor adaptation of distinctive perturbations does not allow for simple switching between motor commands during randomly changing perturbations when they are distinguished by visual cues [18, 19]. On the other hand, subjects relying on reward prediction error (either end-point error or binary reward) were able to easily adapt to the random direction of perturbation. Similar results were found in studies that showed dual adaptation was possible primarily through explicit strategies [20, 21]. Interestingly, the analysis of the inter-subject variance showed that the EPE and RWD groups had smaller variance between subjects compared to the ERR group. This might be because the explicit information on the direction of perturbation with the cues encouraged explicit strategies even in the ERR group for some subjects. Explicit motor learning can be considered as a conscious process that serves as a basis for strategic corrections and doesn’t influence the internal model [17, 22]. This suggests that explicit learning allows for quicker adaptation of motor behaviour. However, this only applies in cases when cues are indirect, meaning they are not part of the dynamic state of the body or manipulated object. In cases where cues are direct, such as the use of separate hands [23, 24], movement locations [25], visual workspace locations [26], or movement plans [27–30], sensorimotor adaptation allows for switching between motor commands during randomly changing perturbations.

The sensorimotor adaptation and explicit reward learning processes cannot be separately observed in human motor learning due to the inability to perfectly separate them or inhibit one of them. To investigate the individual contributions of either sensorimotor adaptation or explicit reward learning on its own, we simulated different learning processes separately in a computational model. During simulation of sensorimotor adaptation, internal model adapts to the new perturbation, resulting in new motor commands. The changes to the motor commands are on the level of an internal model and can be looked at as changed motor commands for a movement resulting in a straight cursor trajectory. The same commands are then used in the case when perturbation is no longer present. Even if the model considers the lack of perturbation, the motor commands do not change since the model treats them as correct for a straight movement. Differently, explicit reward learning (either based on end-point error or binary reward) does not affect the internal model and does not change the motor commands directly. By using the reward error for the whole movement in the next trial, it in effect changes the direction of movement or rather it plans the movement towards a different target. This coincides with experiments that show subjects were aiming in the different direction and knew their hand position was changed [1]. Overall, the simulation results show how the learning proceeds regardless of the prediction error as well as it shows how the aftereffects are dependent on the sensory prediction error and do not occur during explicit reward learning.

Our experimental results clearly show two distinct processes of motor learning with different retention rates. It has been previously proposed that motor learning is comprised of two interacting processes, a fast process that adapts and decays quickly and a slower process that adapts and decays more gradually [30, 31]. Computationally, this was described by a dual-rate model that incorporates different timescales and retention rates [31]. A dual-rate model does not address implicit and explicit learning directly; however, it has been suggested that the fast and slow processes of the dual-rate model may correspond to the explicit and implicit components of learning [32]. A dual-rate model is a single context model and cannot explain the differences between the catch and the de-adaptation trials as well as the differences in switching between perturbations that were observed in our experiment [32]. In contrast, our computational model accounts for the different timescales and retention rates with sensorimotor and reward learning and shows how multiple-contexts (visual cues) have different effects during sensorimotor or reward learning. It is also able to account for differences in catch and de-adaptation trials. Moreover, current models of motor learning usually rely on either sensory prediction error or reward prediction error [33]. However, recent behavioural experiments are showing that different learning processes respond to distinct error signals [1]. Computational models that aim to represent human motor learning as a whole should take into account both sensorimotor adaptation based on sensory prediction error and explicit reward learning based on reward prediction error. The advantage of our computational model is that it separately accounts for sensorimotor and reward prediction errors.

Taking from both the experimental results and the results from the simulation, we argue that only sensorimotor adaptation affects internal model which produces an aftereffect in cases when a person is aware of the lack of perturbation. The changes to the internal model that occurred are not consciously perceived so the person does not know that their hand is moving in another direction. Therefore, when the perturbation is removed, they cannot instantaneously make changes to the internal model in order to compensate for the change in perturbation. On the other hand, explicit reward learning does not affect the internal model, but rather the subjects consciously adapt the direction in which they aim. Therefore, they are aware of the changes to their movement and have a better understanding of where the real position of the hand actually is. When they are informed of the change in perturbation, they can thus consciously change the direction of the movement towards the right target.

Even though the experiment could not completely separate explicit reward learning and sensorimotor adaptation, the simulation allowed us to look at both separately and compare them. We simulated motor learning with ILC where trials were updated from iteration to iteration. Even though we successfully simulated the majority of the trials, it ended up not being the optimal solution. In ILC, error is summed over time-steps so contribution of a basic feed-forward controller results in a curved cursor trajectory even when feedback contribution is omitted. Alternatively, if the changes would be made to the controller and not directly to the motor commands as in ILC, the planned trajectory would also take into account the contributions of the previous steps. This would allow for a better adaptation in the case, where the change in the first step affects the later steps so that no additional change is necessary. Additionally, the schematic representation of the learning process with ILC is not the best representation of the learning processes taking place in the human brain, because it directly modifies the motor commands instead of the feedback motor commands, parameters of the feedforward internal model and the planned motion. A more sophisticated adaptive control model based on the same concept as the simple ILC model used to clarify sensorimotor and reward learning processes could deal with these [34]. It is important for the future work to also include comparing the model to standard models of motor adaptation.

Our results support that the sensorimotor adaptation and explicit reward learning work through distinct adaptation processes. This suggest that only sensorimotor adaptation makes changes to the internal model, while explicit reward learning uses strategies that do not alter internal model and thus do not produce aftereffects. Additionally, we could show that when humans learn motor tasks, they use both learning processes to successfully adapt to the new environment. This contributes to the understanding of human motor adaptation and the importance of separately looking at the sensorimotor adaptation and explicit reward learning, as well as the importance of taking into account both learning processes since humans tend to use a combination of both.

## IV. Methods

### A. Subjects

Twenty-four right-handed volunteers (6 females and 18 males) participated in the study. They were divided in three groups, the ERR group (n = 8, 2 females and 6 males), the EPE group (n = 8, 2 females and 6 males) and the RWD group (n = 8, 2 females and 6 males). Their average age was 27.34 ± 6.61 years, height 178.00 cm ± 10.25 cm and body mass 72.52 ± 15.85 kg. Prior to their participation, the subjects were informed about the course of the experiment and signed a written consent approved by the Slovenian National Medical Committee (No. 339/2017/7). We have pseudonymized all the data obtained from experiments. All experimental protocols were approved by the National Medical Ethics Committee Slovenia (No. 339/2017/7) and the methods were carried out in accordance with the relevant guidelines.

### B. Experiment

Subjects were seated in a chair in front of a table with an integrated screen (Fig 1A). They held the handle of a haptic robot (Haptic Master MK2, Moog, Nieuw-Vennep, The Netherlands) that was located underneath the table and not visible to the subjects. The subjects were asked to perform a series of arm reaching movements. The position of the subject’s hand was displayed on the screen as a cursor.

The ERR group was provided with both types of prediction errors by showing them the cursor during the whole reaching task. Here we assumed that the subjects primarily rely on the sensory prediction error since in the presence of sensory information subjects predominantly disregard the information of the task success [1]. The EPE and RWD groups had the cursor hidden to them during the motion. The EPE group received the information on where they passed the distance of the target (end-point error) and whether the target was hit or missed. The RWD group only received the information whether the target was hit or missed. Since there was no visual feedback during the movement, information on sensory prediction error was minimized and subjects were only able to rely on information regarding the task success (reward prediction error).

There were two types of trials: unperturbed and perturbed. During the unperturbed trials, the motion of the cursor was aligned with the actual movement of the hand while during the perturbed trials the cursor underwent a rotation of 30° in either clockwise (right direction of perturbation) or counter clockwise direction (left direction of perturbation). In effect, when the hand moved straight ahead, the cursor moved either 30° towards the right or towards the left.

A trial started with the Haptic Master positioning the handle to a start position displayed as a white circle with a diameter of 0.8 cm. The target towards which the subjects had to perform the arm movement was a blue circle with a diameter of 1.4 cm which was located 10 cm away from the start position. On the top of the screen, there was a graphical perturbation indicator that displayed the direction of the perturbation in the form of visual cues (straight line for unperturbed trials, right arrow for right direction and left arrow for left direction of perturbation). The visual cues were used because we wanted to measure feedforward learning and not feedback response. Visual cues are used for all trials and, with the exception of the catch trials, they always reflect the true perturbation. Subjects were told that there would be trials without perturbation and trials with perturbation where the position on the screen would not accurately reflect the position of their hand. They were informed that the visual cues will be shown before each trial. They were only given the explanation that visual cues show the direction of perturbation, however no further information on the type of perturbation was given. Subjects were informed about the removal of the perturbation using a corresponding visual cue. Furthermore, they were reminded to pay attention to the perturbation indicators a few trials before the start of the de-adaptation trials.

### C. Protocol

The experiment was divided into two consecutive parts as shown by the two rows on Fig 1B. In the first part, the direction of perturbation was consistent, while in the second part, the direction of perturbation was random.

#### Part 1: Consistent direction of perturbation

The first part with the consistent direction of perturbation was meant for subjects to adapt to a new perturbation. Subjects first learned to adapt to one perturbation and then proceeded with the other direction of perturbation. Half of the subjects started with clockwise rotation and the other half started with the counter clockwise rotation.

Subjects first performed 10 baseline trials without perturbation, followed by 50 trials with perturbation in a single direction. To assess the adaptation to perturbation, we included 2 catch trials near the end of the perturbed trials. Each catch trial was followed by 4 perturbed trials. The first part concluded with 10 de-adaptation trials without perturbation (upper part of Fig 1B).

#### Part 2: Random direction of perturbation

The second part of the experiment was designed to measure adaptation to the randomly changing direction of the perturbation. It started with 20 perturbed trials with clockwise rotation followed by 20 perturbed trials with the counter clockwise rotation. Afterwards, the subjects had to perform a randomly ordered set of 100 trials with either the clockwise or counter clockwise rotation (lower part of Fig 1B). The pattern of the perturbation directions was consistent for all subjects.

### D. Data analysis

Position of the hand during the arm reaching motion was recorded by the haptic robot with a frequency of 30 Hz. Motion data was filtered using a 4^th^ order low-pass Butterworth filter with 10 Hz cut-off frequency. In addition to the hand movement trajectories, we also calculated the cursor trajectories based on the visuomotor rotation that was applied in each trial. To observe motor learning, cursor trajectories were segmented into individual trials where the start of the trial was defined as the moment when the movement trajectory crossed the 0.4 cm target distance relative to the starting position and the end of the trial was defined as the moment when movement trajectory crossed the 10 cm distance relative to the starting position. To assess the effects of perturbation on the motion and the adaptation to perturbation over the trials, we calculated Cursor path area for each trial. Cursor path area is defined as the area between the cursor trajectory and a straight line between the start position and the target and takes into account the whole motion [35]. In cases where the target is not hit the Cursor path area is defined as the area between the cursor trajectory, the straight line between the start position and the target and a circle around the start area with a diameter of 10 cm. To assess the adaptation without the contribution of sensory feedback, we calculated Cursor angle [36] defined as an angle between the y-axis of the coordinate frame and the vector between the cursor position at the beginning of the motion when the velocity reached 0.025 m/s and the cursor position 160 ms after the beginning of the motion. For the first part of the experiment, both the Cursor path area and Cursor angle during the counter clockwise rotation were multiplied with -1, so that the values were comparable to those obtained during clockwise rotation and could be averaged.

To compare ERR, EPE and RWD groups, we compared average trial times of individual subjects, Cursor path area and Cursor angle values of catch trials, Cursor angle values of 1^st^ de-adaptation trials, and average Cursor angle values of individual subjects for trials during random direction of perturbation between the three groups. ANOVA was used for all comparisons between the groups and additional post hoc t-tests with Bonferroni correction were conducted to determine the significant differences between the specific groups. Paired t-test was used to compare average Cursor angle values of the first 10 trials to the average Cursor angle values of the last 10 trials of each individual subject. To test equality of variances we used Bartlett test and additional post hoc F-tests of equality of variances with Bonferroni correction.

### E. Computational model

We modelled the experimental system of a human arm reaching towards a target as seen in the experimental study. The human arm was modelled as a point mass with the mass *m* and described in polar coordinate system with the radial distance *d* and polar angle γ with respect to the initial position in the pole. Our system was designed as a discrete-time, linear time-invariant multiple inputs multiple outputs (MIMO) system

$$x_{j}\left(k+1\right)=\text{A}x_{j}\left(k\right)+\text{B}u_{j}(k)$$ (1)

$$y_{j}\left(k\right)=x_{j}\left(k\right)+r_{j},$$ (2)

where *k* ∈ {1, …, *k*_*end*_} is the time step and *j* ∈ {1, …, *j*_*end*_} is the simulation trial index. Moreover, ***u***_*j*_(*k*) is the control vector

$$u_{j}\left(k\right)=\left[\begin{array}{c} f_{j}^{d}\left(k\right)\\ f_{j}^{\gamma }\left(k\right) \end{array}\right],$$

with $f_{j}^{d}(k)$ being the control force in the radial direction and $f_{j}^{\gamma }(k)$ in the polar angle direction. ***x***_*j*_(*k*) is the state vector defined as

$$x_{j}\left(k\right)=\left[\begin{array}{c} \begin{array}{c} d_{j}\left(k\right)\\ {\dot{d}}_{j}\left(k\right) \end{array}\\ \begin{array}{c} \gamma_{j}\left(k\right)\\ {\dot{\gamma }}_{j}\left(k\right) \end{array} \end{array}\right],$$

and the output ***y***_*j*_(*k*) is defined similarly. ***r***_*j*_ is a rotation vector

$$r_{j}=\left[\begin{array}{c} \begin{array}{c} 0\\ 0 \end{array}\\ \begin{array}{c} \beta \\ 0 \end{array} \end{array}\right],$$

where *β* is the angle of rotation that can either be 0 when there is no visuomotor rotation or ±30º for clockwise and counter clockwise visuomotor rotation. The state matrix is defined as

$$\text{A}=\left[\begin{array}{cc} \begin{array}{cc} 0 & 1\\ 0 & 0 \end{array} & \begin{array}{cc} 0 & 0\\ 0 & 0 \end{array}\\ \begin{array}{cc} 0 & 0\\ 0 & 0 \end{array} & \begin{array}{cc} 0 & 1\\ 0 & 0 \end{array} \end{array}\right]$$

and the input matrix as

$$\text{B}=\left[\begin{array}{cc} \begin{array}{c} 0\\ 1/m \end{array} & \begin{array}{c} 0\\ 0 \end{array}\\ \begin{array}{c} 0\\ 0 \end{array} & \begin{array}{c} 0\\ 1/md^{2} \end{array} \end{array}\right].$$

Performance of the system is determined by the error vector ***e***_*j*_(*k*) in both position and velocity:

$$e_{j}\left(k\right)=\left[\begin{array}{c} \begin{array}{c} e_{j}^{d}\left(k\right)\\ e_{j}^{\dot{d}}\left(k\right) \end{array}\\ \begin{array}{c} e_{j}^{\gamma }\left(k\right)\\ e_{j}^{\dot{\gamma }}\left(k\right) \end{array} \end{array}\right].$$

It is defined as a difference between the desired trajectory and the actual trajectory and is calculated as

$$e_{j}\left(k\right)=y^{d}\left(k\right)-y_{j}\left(k\right)+n_{s},$$ (3)

where ***y***^*d*^(*k*) is the desired trajectory, ***y***_*j*_(*k*) is the actual trajectory and ***n***_s_ is a normally distributed sensory noise with variance $\sigma_{d}^{2}=0.002$ for distance, $\sigma_{\gamma }^{2}=0.3$ for angle, $\sigma_{\dot{d}}^{2}=0.004$ for velocity and $\sigma_{\dot{\gamma }}^{2}=0.05$ for angular velocity. End-point error for explicit reward learning is calculated as

$$e_{j}^{RW}=\sum_{l=1}^{k_{end}}(2e_{j}^{\gamma }(l)∙d_{j}(l))/k_{end}$$ (4)

and is the same for all time steps. In cases where only end-point information is provided and any sensorimotor information during the trial is omitted, this results in

$$e_{j}^{RW}=2e_{j}^{\gamma }\left(k_{end}\right)∙d_{j}\left(k_{end}\right).$$ (5)

In cases where the end-point error is not available and reward information is only binary, error for explicit reward learning is calculated as

ejRW=0ifejRW≤r1ifejRW>r, (6)

where *r* is the radius of the target.

To model the human learning process, we used a non-causal iterative learning control (ILC) system, where a current-iteration feedback controller is incorporated with ILC in the parallel architecture and combined with learning based on reward prediction error as seen on Fig 4. We modelled the human learning process by combining the input from the explicit reward learning $u_{j}^{RW}(k)$, input from the feedforward sensorimotor adaptation $u_{j}^{SM}(k)$ and input from the feedback control $u_{j}^{FB}(k)$, into the motor command

$$u_{j}\left(k\right)=u_{j}^{RW}\left(k\right)+u_{j}^{SM}\left(k\right)+u_{j}^{FB}\left(k\right).$$ (7)

The model assumes an additive effect of sensorimotor adaptation and explicit reward learning during a single trial, however, the amount of explicit reward learning has an effect on the sensorimotor learning in the succeeding trials.

Learning based on reward prediction error is used to plan the direction of movement. It either takes into account the difference between the actual and the optimal movement of the cursor in the previous simulation trial or explores different movements to determine the movement in the next simulation trial as

$$u_{j}^{RW}\left(k\right)=u_{0}(k)+u_{j-1}^{RW}(k)+p{\;e}_{j-1}^{RW},$$ (8)

Where *p* is an explicit reward learning parameter. In the case of end-point error learning, parameter *p* is set to a fixed value, while during the exploration phase in the binary reward learning parameter *p* is sampled from a uniform distribution. When a successful movement is found, motor commands for this movement are stored in the memory module. ***u***_0_ represents the previous motor knowledge for an unperturbed movement that produces a straight minimum jerk trajectory [37] between the initial point and target. This knowledge is stored in the memory / fallback module and represents the initial conditions of motor commands for the first simulation trial (*j* = 1). Furthermore, these initial conditions are used when the perturbation is removed and the system is aware of the lack of perturbation (de-adaptation trials). It is not used during catch trials, since the system doesn’t know the perturbation is removed.

Learning based on sensory prediction error is used for feedforward sensorimotor adaptation that is updated from one simulation trial to the next as

$$u_{j}^{SM}\left(k\right)=b\left(u_{j-1}^{SM}\left(k\right)+\text{L}\;e_{j-1}\left(k\right)\right)+n_{m},$$ (9)

where *b* is forgetting factor set to 0.995, **L** is a matrix representing the feedforward sensorimotor adaptation parameters and ***n***_m_ is normally distributed motor noise with variance $\sigma_{f^{d}}^{2}=0.02$ for distance and $\sigma_{f^{\gamma }}^{2}=0.8$ for angle.

Input from the feedback control $u_{j}^{FB}(k)$ is updated from one time-step to the next during a single simulation trial as

$$u_{j}^{FB}\left(k\right)=\text{C}\;e_{j}\left(k-1\right),$$ (10)

where **C** is a matrix representing feedback sensorimotor parameters. We incorporated a delayed reaction time of 0.3 s for feedback control, based on the experimental data.

The described model is used to carry out simulations of the reaching task described in above Experiment section. There were 40 time steps of 0.02 s each. To allow for a direct comparison between the experimental and simulation results, the number of simulation trials was set to 80 and the presence of visuomotor rotation was included in the same number of simulation trials as in the experiment. Simulations included the baseline trials, trials with perturbation in one direction, 2 catch trials and deadaptation trials (as shown in the top part of Fig 1B).

Parameter *p* and parameters in matrices **L** and **C** model the learning rates and were changed for different simulation runs. The presented results include a range of different values between maximum and minimum parameter values where the model was stable. *p* models the learning rate of the explicit reward learning in the case of end-point error learning and also models the exploration in the case of binary reward learning, while **L** models the learning rate of the sensorimotor adaptation. To investigate the end-point error learning process, we carried out simulation runs where we modulated the parameter *p* while **L** was set to $\left[\begin{array}{cc} \begin{array}{cc} 0 & 0 \end{array} & \begin{array}{cc} 0 & 0 \end{array}\\ \begin{array}{cc} 0 & 0 \end{array} & \begin{array}{cc} 0 & 0 \end{array} \end{array}\right]$. To investigate binary reward learning process, we carried out simulation runs where parameter *p* was sampled from uniform distribution while **L** was again set to $\left[\begin{array}{cc} \begin{array}{cc} 0 & 0 \end{array} & \begin{array}{cc} 0 & 0 \end{array}\\ \begin{array}{cc} 0 & 0 \end{array} & \begin{array}{cc} 0 & 0 \end{array} \end{array}\right]$. On the other hand, to investigate the sensorimotor adaptation process, we carried out simulation runs where we modulated the parameters in matrix **L** while *p* was set to 0. **C** represents the PD feedback controller and was set to $\left[\begin{array}{cccc} 0.4 & 5 & 0 & 0\\ 0 & 0 & 0.4 & 5 \end{array}\right]$ for all simulation runs. Parameter **C** was chosen so that the feedback contribution best matches the experimental results. However, when we simulated trials where no sensory information was provided, there was no contribution of the feedback control due to the absence of sensory prediction error. Additionally, we carried out simulation runs with a combination of both reward and sensorimotor adaptation, to capture the learning processes observed in the experimental study. Here, the parameter values were chosen so that the model best captures the experimental results. To obtain representative values, we ran the simulations for each combination of parameter values 50 times and then calculated the average values.
